# Fluorinated copolymer resists for high‐resolution negative‐tone lithography: Preparation and patterning performance

**DOI:** 10.1002/smo2.70062

**Published:** 2026-05-28

**Authors:** Yuting Tang, Wenzheng Li, Xinyan Huang, Min Zhang, Han Han, Jialong Zhang, Qinyu Luo, Jiangli Fan, Pengzhong Chen, Xiaojun Peng

**Affiliations:** ^1^ State Key Laboratory of Fine Chemicals, Frontiers Science Center for Smart Materials Dalian University of Technology Dalian China; ^2^ Sinopec Shanghai Research Institute of Petrochemical Technology Co., Ltd. Shanghai China

**Keywords:** electron‐beam lithography, fluorinated methacrylate polymers, photoresists, radical polymerization

## Abstract

In response to the increasing demands in advanced lithography for high‐resolution and highly stable photoresists, the development of high‐performance resist materials has become critically important. In this study, a series of poly (tert‐butyl methacrylate‐*co*‐adamantyl methacrylate‐*co*‐[trifluoroethyl methacrylate/1,1,1,3,3,3‐hexafluoroisopropyl isobutyrate methacrylate]) copolymers (PTAF) were rationally designed and synthesized, and their preparation conditions were systematically optimized via a single‐factor experimental design. The molecular weight distribution, thermal properties, hydrophobicity, and electron‐beam lithography (EBL) performance of the resulting polymers were comprehensively investigated. The results show that PTAF exhibits a narrow molecular weight distribution (polydispersity index = 1.2–1.3) and a number‐average molecular weight (*M*_*n*_) of approximately 4271 g mol^−1^. The polymer demonstrates good thermal stability, with an initial decomposition temperature (*T*_d_) of 212°C and a glass transition temperature (*T*_g_) of 105°C, along with pronounced hydrophobicity. EBL results reveal that PTAF displays intrinsic negative‐tone behavior in the absence of externally added photoacid generators, enabling fabrication of high‐resolution patterns with a minimum linewidth of 16.5 nm. Mechanistic studies indicate that fluorinated monomers undergo ionization upon irradiation, initiating in situ acid generation, which subsequently triggers the deprotection of tert‐butyl ester groups during post‐exposure baking. This process leads to a polarity reversal and a corresponding solubility switch in the resist system. Overall, this work establishes a high‐performance ternary copolymer photoresist platform and elucidates its negative‐tone imaging mechanism, providing valuable insights for the design of polymer‐based resists in advanced lithography, particularly for extreme ultraviolet applications.

## Graphical abstract

Electron‐beam induced C–F cleavage in –CF_3_ generates F^−^, which abstracts a proton to form the acid that catalyzes tBMA deprotection during post‐exposure baking. This self‐sensitization mechanism enables the precise formation of high‐resolution negative‐tone patterns with enhanced sensitivity and resolution.

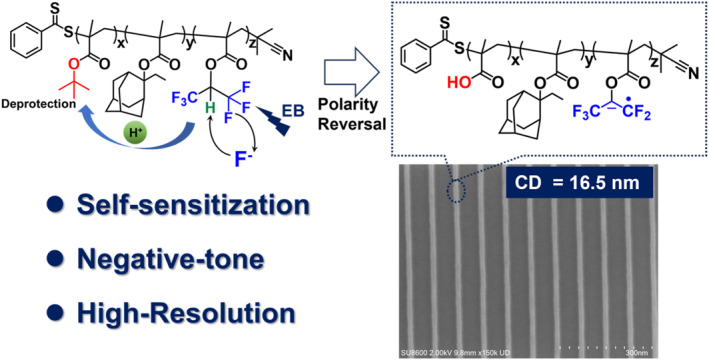

## 1 INTRODUCTION

Lithography is a core technology in microelectronics manufacturing, and its resolution directly determines the integration density and performance of integrated circuits. As the key functional material in the lithographic process, photoresists decisively influence pattern fidelity and overall lithographic performance.[^1^, ^2^, ^3^, ^4^, ^5^] With semiconductor manufacturing advancing to more advanced nodes, photoresist resin materials must satisfy increasingly stringent requirements, including high thermal stability, appropriate hydrophobicity for compatibility with development systems, and the capability to produce high‐resolution lithographic patterns.[^6^, ^7^]

Currently, widely used chemically amplified resists rely on photoacid generators (PAGs) incorporation to achieve high‐sensitivity imaging.^[8]^ However, the physical blending of PAGs can no longer meet the stringent demands of advanced process nodes. This has driven a major research focus toward covalently bonding PAG units to the polymer backbone. For example, the Gao Xiang research group covalently anchored triphenylsulfonium salt 4‐(vinyl) benzenesulfonate (VBS‐TPS) to the resin backbone, achieving micron‐scale patterning.^[9]^ Similarly, the Ober research group developed PAG‐bound self‐immolative polymer resists that achieve a high‐sensitivity of <10 mJ cm^−2^.^[10]^ However, improvements in high‐resolution performance with grafted PAG resins remain limited due to the acid diffusion paradox: while higher PAG content increases sensitivity, it also compromises the resolution.[^11^, ^12^] Consequently, single‐component photoresist systems that eliminate the need for external PAGs have emerged as a promising direction for next‐generation lithographic materials. These systems exploit the intrinsic autocatalytic properties of the resist matrix to generate active species in situ upon irradiation, enabling precise modulation of solubility. Such an approach not only simplifies the material composition but also effectively suppresses acid diffusion, leading to enhanced imaging resolution and improved dimensional fidelity.^[13]^

Methacrylate resins have attracted considerable attention in the field of photoresists because of their high structural tunability, abundant monomer sources, and excellent film‐forming properties. Previous studies have demonstrated that methacrylate polymers exhibit high transparency at advanced lithographic wavelengths (e.g., 193 nm), making them promising alternatives to conventional aromatic polymers.^[14]^ By rationally selecting methacrylate monomers with different structural features, the key properties of photoresist resins can be precisely tailored.[^15^, ^16^, ^17^, ^18^, ^19^] For example, monomers containing adamantane groups can significantly improve the glass transition temperature and etch resistance of the resin, while their hydrophobic nature also regulates polymer solubility in developer solutions and suppresses swelling during development.[^20^, ^21^, ^22^, ^23^]

Fluorinated monomers, owing to the strong hydrophobicity and low surface energy of fluorine atoms, can enhance development selectivity and overall resin hydrophobicity.[^24^, ^25^, ^26^] Guo et al. reported that fluorinated monomers not only increase extreme ultraviolet (EUV) absorption but also generate F^−^ species through C–F bond cleavage to participate in subsequent reactions, thereby enhancing photolithographic sensitivity, highlighting the potential of fluorinated copolymers as next‐generation photoresist materials.^[27]^ Meanwhile, tert‐butyl methacrylate (tBMA) has become a classic structural unit in photoresist resin design due to its excellent compatibility and tunable solubility.^[28]^ Li et al. employed reversible addition–fragmentation chain‐transfer polymerization to synthesize photoresist polymers from four (meth)acrylate monomers.^[29]^ Benefiting from the narrow molecular weight distribution (polydispersity index [PDI] ≤1.32) and good thermal stability (initial decomposition temperature ≥200°C) achieved through controlled polymerization, the resulting materials enabled high‐resolution patterning of 0.18 μm, demonstrating the importance of polymerization control for lithographic performance.

Block polymerization of functional monomers integrates the structural and performance advantages of individual monomer units to construct multifunctional photoresist resins, representing an important research direction for synergistic modification. However, major challenges remain. Most studies focus on binary copolymers or monofunctional monomers modification, whereas methacrylate‐based triblock polymers that simultaneously incorporate multiple functional groups, such as adamantyl, fluorinated, and ester moieties have been rarely investigated. As a result, the synergistic effects of these multifunctional units on the photoresist performance remain unclear. In multi‐monomer block copolymer systems, precise control over molecular weight distribution and structural uniformity through polymerization parameters is difficult. Therefore, matching polymer structure and performance through process optimization is important.

In this study, poly (tert‐butyl methacrylate‐*co*‐adamantyl methacrylate‐*co*‐[trifluoroethyl methacrylate/1,1,1,3,3,3‐hexafluoroisopropyl isobutyrate methacrylate]) copolymers (PTAF) were designed and synthesized using tBMA, 2‐ethyl‐2‐adamantyl methacrylate (EAdMA), trifluoroethyl methacrylate (TFEMA), and 1,1,1,3,3,3‐hexafluoroisopropyl isobutyrate methacrylate (HFIPMA) as monomer units. The preparation process was optimized through single‐factor experiments, and the polymer structure and thermal properties were characterized by proton nuclear magnetic resonance (^1^H NMR), fourier‐transform infrared spectroscopy (FT‐IR), gel permeation chromatography (GPC), thermogravimetric analysis (TGA), and differential scanning calorimetry (DSC). Their molecular weight distribution, hydrophobicity, and photolithographic performance were systematically investigated. The results demonstrate that electron‐beam exposure, the fluorinated polymer generates clear and dense patterned lines with a critical dimension (CD) of 16.5 nm at an exposure dose of 27.5 nC cm^−1^. These findings provide useful guidance for the design of high‐performance photoresist resins and offer valuable technical support for the development of advanced photolithography technologies.

## 2 EXPERIMENTAL

### 2.1 General information and materials

tBMA (97%), EAdMA (97%), TFEMA (97%), HFIPMA (97%), 2‐cyano‐2‐propylbenzodithioate (CPDB, 97%), and azobis (isobutyronitrile) (AIBN, 97%) were purchased from Anajie (Shanghai) Pharmaceutical Chemical Co. Ltd. *N*,*N*‐dimethylformamide (DMF, 99.9%), propylene glycol monomethyl ether acetate (PGMEA, electronic grade), deionized water, *n*‐heptane (99%), tetrahydrofuran (THF, chromatographic grade), and deuterated chloroform (CDCl_3_, 99.8%) were obtained from Shanghai Aladdin Biochemical Technology Co. Ltd. Unless otherwise noted, all reagents and solvents were used as received without further purification.

The chemical structure of the polymers was confirmed by ^1^H NMR spectroscopy using a Bruker Avance II 400 MHz spectrometer (Bruker), with deuterated chloroform (CDCl_3_) as the solvent and tetramethylsilane as the internal standard. FT‐IR spectra were recorded on a ThermoFisher Nicolet 6700 spectrometer (ThermoFisher Scientific) over a wavelength range of 4000–400 cm^−1^ to identify functional groups and monitor chemical changes. The absorption spectra were recorded using a Lambda 35 UV–vis spectrophotometer (PerkinElmer). Polymer molecular weights and distributions were determined using the HLC‐8420GPC EcoSEC Elite gel permeation chromatograph (Tosoh), with chromatographic‐grade THF as the mobile phase and polystyrene standards. The advancing (*θ*_a_) and receding (*θ*_r_) contact angles of the photoresist films were measured using a Dataphysics OCA50 video optical contact angle goniometer (Dataphysics). Thermal transition behavior and stability were evaluated using a DSC3 differential scanning calorimeter (Bruker) and a Mettler Toledo TGA/DSC1/1100SF thermogravimetric analyzer (Mettler Toledo), respectively. The elemental composition and chemical states of the polymer surfaces were analyzed by X‐ray photoelectron spectroscopy (XPS) (ESCALAB Xi+, Thermo Fisher Scientific). Additionally, a Sartorius BCE2241‐1CCN analytical balance (Sartorius) was used for precise weighing of materials, a WS‐650 spin coater (MYCRO) for film preparation, and a Dimension Icon atomic force microscope (Bruker) to examine the surface topography and roughness of the photoresist films.

### 2.2 Photoresist preparation and lithography process

As a representative example, PTAFIII (40 mg) was dissolved in 1 mL of PGMEA and filtered through a 0.22 μm polytetrafluoroethylene membrane to obtain a clear, transparent solution. Thirty‐five microliter of solution was spin‐coated onto a silicon wafer at a specific speed for 30 s. The coated films were pre‐baked at 50°C for 120 s to remove residual solvent, resulting in a uniform photoresist layer. Exposure was carried out using a 254 nm xenon lamp or an electron‐beam lithography (EBL) system. The exposed films were subsequently developed in *n*‐heptane for 30 s to yield the negative photolithography patterned structures.

### 2.3 Exposure testing

Line exposure experiments were conducted to evaluate the resolution limits. The experimental conditions were as follows: contrast measurements were performed at an acceleration voltage of 2 kV using an 8 × 8 matrix of 5 μm square patterns. The exposure dose ranged from 20 to 1280 μC cm^−2^ for PTAI and from 10 to 640 μC cm^−2^ for PTAFII and PTAFIII; an acceleration voltage of 30 kV was used, with exposure doses ranging from 15 to 37.5 nC cm^−1^ and a pitch of 100 nm. *n*‐Heptane was employed as the developer, and the development time was fixed at 30 s. EBL was performed on PTAI, PTAFII, and PTAFIII. The resulting patterned structures were characterized using a Hitachi SU8600 scanning electron microscope combined with a Raith pattern generator (Hitachi; Raith) to assess high‐resolution morphology.

### 2.4 Etch resistance test

Etch resistance tests were conducted using an inductively coupled plasma (ICP) etcher. The photoresist was first spin‐coated onto a clean silicon wafer substrate. After prebaking, the films were exposed via EBL under the following conditions: 30 kV acceleration voltage, 30 nC cm^−1^ dose, and 150 nm pitch. The exposed films were developed in *n*‐heptane to produce well‐defined micro‐ and nano‐patterned structures, which served as the etch test samples. Etching was performed under constant process conditions using SF_6_ plasma, with a gas flow rate of 15 sccm, radio frequency power of 200 W, and etching time of 5 s. The change in photoresist film thickness before and after etching was measured by atomic force microscopy (AFM), and the etching rate was calculated accordingly.

### 2.5 Mechanism study

XPS was employed to investigate the chemical state of the PTAFIII films. Initially, XPS spectra were collected from the unexposed PTAFIII film. The films were subjected to electron beam irradiation at a dose of 300 μC cm^−2^, and XPS measurements were performed on the irradiated samples. The films were then exposed to a xenon lamp equipped with a 254 nm filter for 5, 10, and 30 min, respectively, and XPS measurements were repeated after each exposure. All collected spectra were subjected to peak fitting analysis using Avantage software to determine elemental valence states and atomic concentrations.

## 3 RESULTS AND DISCUSSION

### 3.1 Synthesis and properties of fluorinated copolymers

**FIGURE 1:**
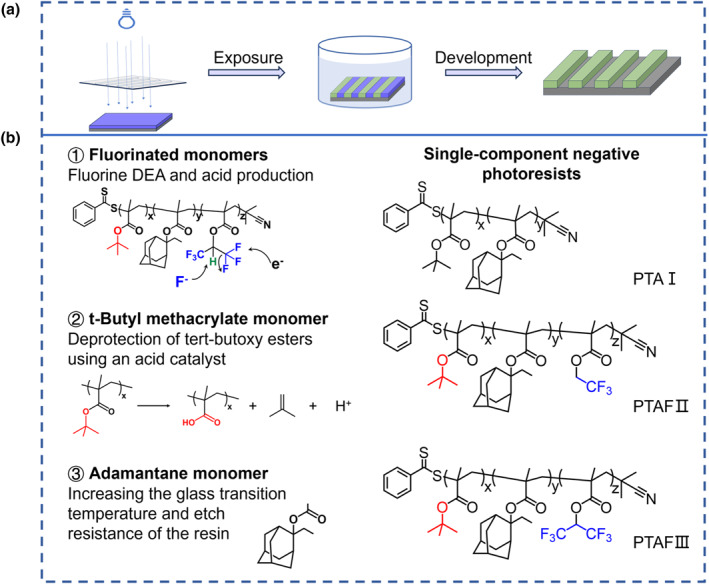
(a) Schematic diagram of the photolithography process, (b) monomer selection and resin structure design.

The three photoresist resins designed in this study are constructed based on a synergistic strategy employing multifunctional methacrylate monomers, whereby the overall photoresist performance is optimized through complementary roles of each component. The pattern transfer process is illustrated in Figure 1a, where the mask pattern is transferred onto the substrate via exposure and subsequent development steps. In terms of monomer selection, fluorinated monomers (TFEMA and HFIPMA) leverage the intrinsic hydrophobicity and low surface energy of fluorine atoms to enhance the hydrophobic character of the resin; meanwhile, they are capable of undergoing acid‐catalyzed deprotection reactions in the presence of dissociative electron attachment (DEA). The adamantane‐based monomer (EAdMA) improves the *T*_g_ and etch resistance owing to its rigid alicyclic structure, while also modulating development solubility and suppressing swelling. In addition, tBMA exhibits excellent compatibility, and its tert‐butyl protecting group undergoes acid‐catalyzed deprotection, providing essential reactive sites for pattern formation. Collectively, these features lead to the formation of a photoresist matrix resin with enhanced overall performance (Figure 1b).

**FIGURE 2:**
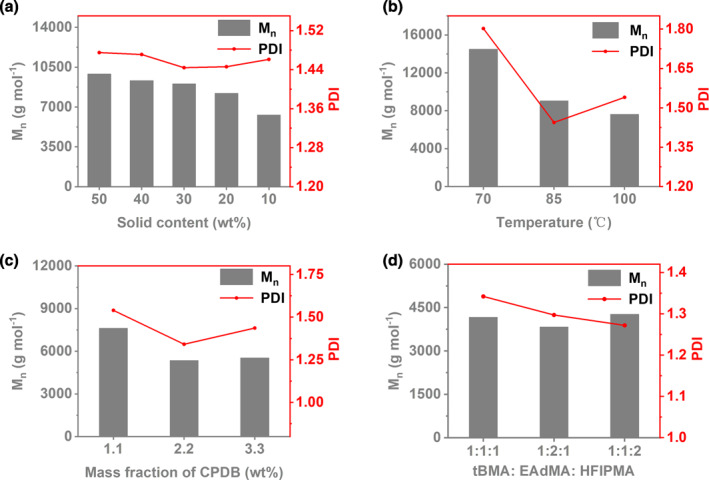
(a) Effect of solid content of the reaction system, (b) polymerization temperature, (c) amount of CPDB, (d) monomer ratio on *M*_*n*_ and PDI. CPDB, 2‐cyano‐2‐propylbenzodithioate; PDI, polydispersity index.

Using tBMA, EAdMA and HFIPMA as monomers, AIBN as the initiator and CPDB as the chain transfer agent, the effects of solid content (Figure 2a, Supporting Information S1: Table S1), reaction temperature (Figure 2b, Supporting Information S1: Table S2), CPDB content (Figure 2c, Supporting Information S1: Table S3) and monomer ratio (Figure 2d, Supporting Information S1: Table S4) on the *M*_*n*_ and PDI of the copolymers were investigated. Specifically, a decrease in solid content resulted in a reduction of *M*_*n*_, with PDI initially decreasing and then increasing. The optimal concentration was found to be 30 wt%. This phenomenon can be attributed to the synergistic effects of DMF, which provides dilution, solvation, and chain transfer reactions. The reaction temperature, as a crucial thermodynamic parameter, governs the initiator decomposition, the active chain propagation, and the efficiency of chain‐transfer and termination reactions. Consequently, *M*_*n*_ decreased with increasing temperature, while PDI exhibited a decreasing‐then‐increasing trend; the optimal temperature was identified as 85°C. The concentration of the chain transfer agent CPDB regulated the growth and termination pathways of the active chains. With increasing CPDB loading, both *M*_*n*_ and PDI initially decreased and then increased. The optimal control over molecular weight and distribution was achieved with 2.2 wt% CPDB. Moreover, increasing the concentration of the fluorinated monomer HFIPMA enhanced the consistency of chain growth, improved the homogeneity of the system, and reduced the likelihood of chain transfer and radical termination, leading to a gradual decrease in PDI. When the monomer ratio of tBMA:EAdMA:HFIPMA was set to 1:1:2, the molecular weight distribution reached its optimal state.

These results indicate that the minimum PDI was obtained under the optimized conditions below: 30 wt% solid content, 85°C polymerization temperature, 2.2 wt% CPDB, and a tBMA:EAdMA:HFIPMA molar ratio of 1:1:2. Based on these optimal conditions, a series of copolymers, PTAI‐PTAFIII, were further synthesized (Supporting Information S1: Scheme S1). The key parameters, including the monomer feed ratio, *M*_*n*_, *M*_*w*_, and PDI are summarized in Table 1.

**TABLE 1:** Reaction temperature, feed ratio, *M*_*n*_, *M*_*w*_, and PDI of the copolymers.

Copolymers	Temperature (°C)	Molar feed ratios				*M*_*n*_ (g mol^−1^)	*M*_*w*_ (g mol^−1^)	PDI
		tBMA	EAdMA	TFEMA	HFIPMA			
PTAI	85	50	50	–	–	3300	4140	1.255
PTAFII	85	25	25	50	–	3830	4970	1.297
PTAFIII	85	25	25	–	50	4271	5435	1.272

Abbreviations: EAdMA, 2‐ethyl‐2‐adamantyl methacrylate; HFIPMA, 1,1,1,3,3,3‐hexafluoroisopropyl isobutyrate methacrylate; PDI, polydispersity index; PTAF, poly (tert‐butyl methacrylate‐*co*‐adamantyl methacrylate‐*co*‐[trifluoroethyl methacrylate/1,1,1,3,3,3‐hexafluoroisopropyl isobutyrate methacrylate]) copolymers; tBMA, tert‐butyl methacrylate; TFEMA, trifluoroethyl methacrylate.

GPC characterization confirmed that all copolymers were synthesized under the aforementioned optimized reaction conditions featuring both *M*_*n*_ and *M*_*w*_ in the range of 3300–6000 g mol^−1^, with PDI stably maintained at 1.2–1.3. These results verify the excellent controllability of the developed radical polymerization process.

**FIGURE 3:**
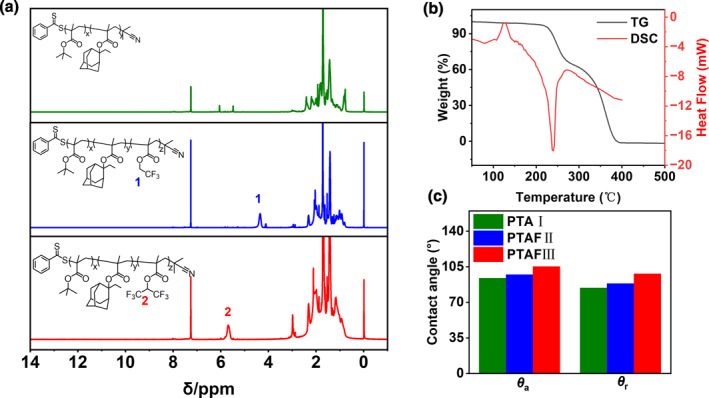
Synthesis and properties of the copolymers. (a) ^1^H NMR spectra of the copolymer, (b) TG and DSC curves of PTAFIII, (c) *θ*_a_ and *θ*_r_ of different polymers. DSC, differential scanning calorimetry; PTAF, poly (tert‐butyl methacrylate‐*co*‐adamantyl methacrylate‐*co*‐[trifluoroethyl methacrylate/1,1,1,3,3,3‐hexafluoroisopropyl isobutyrate methacrylate]) copolymers.

The structures of PTAI, PTAFII and PTAFIII were characterized using ^1^H NMR, FT‐IR and UV–vis spectroscopy (Figure 3a, Supporting Information S1: Figures S1–S5).^[30]^ The characteristic peak at 5.68 ppm originates from the proton linked to the –CF_3_ group in TFEMA, and the peak at 4.35 ppm is assigned to the proton in HFIPMA. These distinct signals directly confirm the successful incorporation of fluorinated monomers. The thermal behavior of PTAFIII was analyzed via TGA and DSC (Figure 3b). PTAFIII exhibited an initial decomposition temperature of approximately 212°C, and *T*_g_ of 105°C, demonstrating high thermal stability that fully satisfies the thermal baking temperature requirements of the standard photolithography process.

To evaluate the surface wettability and hydrophobicity, which are critical for developer compatibility and pattern fidelity in lithography, contact angle measurements were performed on the copolymer films (Figure 3c). The fluorinated copolymers exhibited significantly higher advancing and receding contact angles than the non‐fluorinated ones. The advancing contact angle increased from 93.78° to 105.25° and the receding contact angle rose from 84.24° to 98.05°. Both contact angles increased with the introduction of fluorine atoms. This enhancement in hydrophobicity can be attributed to the strong electronegativity of fluorine, which generates highly polar C–F bonds. Furthermore, the fluorinated side chains preferentially segregate and arrange compactly at the polymer surface, forming a dense hydrophobic layer with low‐surface‐energy.

### 3.2 Photolithographic properties of fluorinated copolymers

**FIGURE 4:**
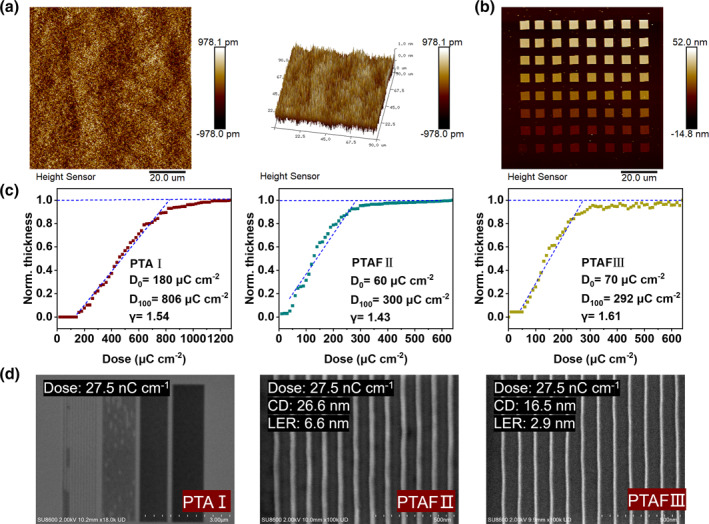
Lithographic performance of the copolymers. (a) AFM 2D and 3D morphology images of PTAFIII, (b) AFM 2D image of PTAFIII after performing an 8 × 8 square array EBL exposure at an accelerating voltage of 2 kV and developing with *n*‐heptane, (c) contrast curves of photoresists PTAI, PTAFII, and PTAFIII after electron beam exposure at different doses (10–1280 μC cm^−2^), (d) SEM images of PTAI, PTAFII, and PTAFIII after EBL exposure at a dose of 27.5 nC cm^−1^. AFM, atomic force microscopy; EBL, electron‐beam lithography; PTAF, poly (tert‐butyl methacrylate‐*co*‐adamantyl methacrylate‐*co*‐[trifluoroethyl methacrylate/1,1,1,3,3,3‐hexafluoroisopropyl isobutyrate methacrylate]) copolymers.

AFM was employed to characterize the surface roughness of copolymer photoresist films and film thickness (Supporting Information S1: Figures S6 and S7). Among them, PTAFIII exhibited the lowest surface roughness, with a root‐mean‐square roughness (Rq) of 0.311 nm, indicating a continuous, dense and defect‐free film surface (Figure 4a). The excellent film uniformity provides a solid foundation for high‐precision photolithographic pattern transfer during exposure.

Subsequently, an 8 × 8 square array exposure was carried out via an electron beam at an acceleration voltage of 2 kV (Figure 4b). The lithographic contrast curve was plotted with the exposure dose as the *x*‐axis and the normalized residual film thickness after development as the *y*‐axis, where *D*_0_ represents the critical exposure dose required for the formation of a negative‐tone pattern formation, while *D*_100_ denotes the exposure dose a required to reach the maximum film thickness (Figure 4c). The contrast (γ) was calculated using the formula $\gamma ={|\mathrm{lg}\left(\frac{D_{100}}{D_{0}}\right)|}^{-1}$. The results are summarized as follows: for PTAI, *γ* = 1.54, *D*_0_ = 180 μC cm^−2^, and *D*_100_ = 806 μC cm^−2^; for PTAFII, *γ* = 1.43, *D*_0_ = 60 μC cm^−2^, and *D*_100_ = 300 μC cm^−2^; for PTAFIII, *γ* = 1.61, *D*_0_ = 70 μC cm^−2^, and *D*_100_ = 292 μC cm^−2^. Clearly, the fluorinated polymers exhibit higher sensitivity than the non‐fluorinated resist.

Line exposure was employed to evaluate the resolution limits of the prepared photoresist. The effects of key photolithography process parameters on the patterning performance were systematically investigated (Supporting Information S1: Figures S8–S11). The results show that optimal resolution was achieved at a resist concentration of 40 mg mL^−1^, a pre‐bake temperature of 50°C, a spin‐coating speed of 6000 rpm, and a post‐bake temperature of 70°C. EBL exposures were subsequently carried out on PTAI, PTAFII, and PTAFIII at an exposure dose of 27.5 nC cm^−1^ (Figure 4d). PTAI failed to form clear patterned lines, while PTAFIII achieved the highest resolution with CD of 16.5 nm and a line edge roughness (LER) of 2.9 nm. This remarkable difference can be explained by the lack of fluorine in PTAI, which limits the regulation of polarity and solubility switching. As a result, the solubility contrast after exposure hinders pattern formation. In contrast, the high fluorine content in PTAFIII strengthens the electron‐withdrawing effect and enhances the photoreaction efficiency. This increase in reactivity amplifies the solubility contrast, reduces edge roughness and pattern collapse, and ultimately enables superior high‐resolution patterning.

**FIGURE 5:**
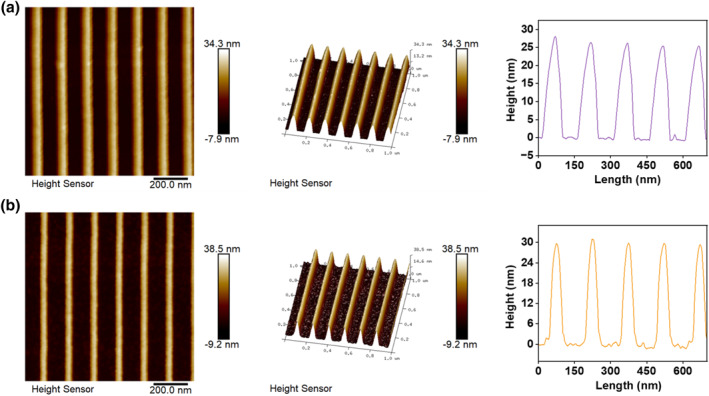
AFM 2D and 3D morphology images and thickness comparison of PTAFIII after electron beam exposure before and after plasma etching treatment. (a) Before etching, (b) after etching. AFM, atomic force microscopy; PTAF, poly (tert‐butyl methacrylate‐*co*‐adamantyl methacrylate‐*co*‐[trifluoroethyl methacrylate/1,1,1,3,3,3‐hexafluoroisopropyl isobutyrate methacrylate]) copolymers.

The etch resistance of the exposed photoresist patterns was evaluated using an ICP etcher with SF_6_ plasma. The etching rate of silicon under the same etching conditions, the etching rate of bare silicon was recorded as 7 nm s^−1^.^[31]^ The film thickness of PTAFIII photoresist was measured by AFM before and after etching, yielding values of 26.8 and 30.8 nm, respectively (Figure 5). The etching rate of PTAFIII was calculated to be 6.09 nm s^−1^, corresponding to an etch selectivity ratio of 1.15 relative to silicon. The 2D and 3D AFM topographic images reveal that the patterned structures remain intact and morphologically stable after plasma etching.

### 3.3 Radiation‐induced lithography mechanisms

**FIGURE 6:**
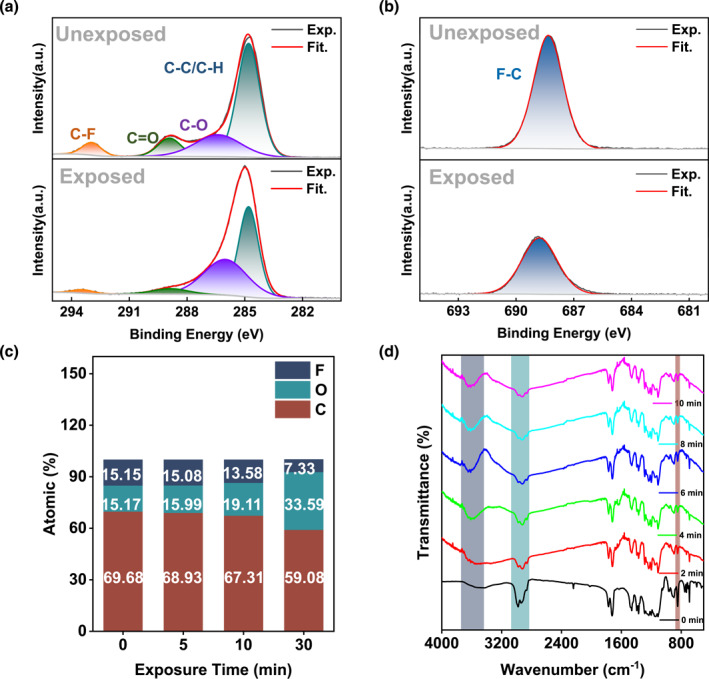
Characterization of the chemical structure and elemental composition of the photoresist before and after irradiation: (a) C 1s XPS spectra before and after EBL exposure; (b) F 1s XPS spectra before and after EBL exposure; (c) histogram of C, O, and F content before and after 254 nm irradiation; (d) FT‐IR spectra before and after 254 nm irradiation. EBL, electron‐beam lithography; FT‐IR, fourier‐transform infrared spectroscopy; XPS, X‐ray photoelectron spectroscopy.

To investigate the chemical reactions of the photoresist upon irradiation, XPS and FT‐IR spectroscopy were used to characterize the photoresist films before and after exposure (Figure 6, Supporting Information S1: Figure S12 and Table S5). In the C 1s XPS spectra (Figure 6a), the binding energies (*E*_b_) for the different carbon species are as follows: C–C/C–H at 284.8 eV, C–O at 286.01 eV, C=O at 288.85 eV, and C–F at 293.41 eV. Calculations reveal that, compared to the unexposed sample, the peak intensities of the exposed sample shows: a 7.33% increase in C–C/C–H (from 31.52% to 38.85%), a 5.97% decrease in C=O (from 10.14% to 4.17%), a 9.74% increase in C–O (from 24.10% to 33.84%), and a 2.33% decrease in C–F (from 3.92% to 1.59%). These changes suggest the cleavage of the C–F bond and the deprotection of the tert‐butyl group. The F 1s spectra (Figure 6b) also reveal a significant decrease in the peak intensity after exposure. Calculations indicate that the F–C content in the exposed sample decreased by 4.00% (from 15.15% to 11.15%), supporting the conclusion of C–F bond cleavage. UV irradiation experiments at 254 nm were performed on PTAFIII samples. The XPS results demonstrate that, with increasing irradiation time, the elemental composition of the photoresist film undergoes pronounced time‐dependent evolution. This behavior is consistent with the trends observed under electron beam exposure, as discussed above (Figure 6c). Specifically, the proportion of carbon decreases by 10.6% (from 69.68% to 59.08%), while the proportion of fluorine decreases by 7.82% (from 15.15% to 7.33%). This suggests that the deprotection of the tert‐butyl ester monomer leads to carbon loss, while the C–F bonds in the trifluoromethyl groups undergo photochemical cleavage.

To investigate the changes in the chemical structure of PTAFIII under UV irradiation, the samples were irradiated with 254 nm light for exposure times of 2, 4, 6, 8, and 10 min, followed by infrared spectroscopy analysis (Figure 6d). The intensity of the absorption peak at 780 cm^−1^ (C–F bond) increased with increasing exposure time, indicating the cleavage of the C–F bonds. This observation corroborates the XPS data. Additionally, the characteristic peak at 2986 cm^−1^ gradually declines while the absorption peak at 3640 cm^−1^ (hydroxyl group) intensifies, consistent with acid‐catalyzed deprotection and polarity conversion. To evaluate its acid‐generating capability, PTAFIII polymer films were doped with the pH indicator bromophenol green, and the UV absorption spectra were recorded before and after UV irradiation at 254 nm (Supporting Information S1: Figure S13). Following exposure, a pronounced decrease in the absorption intensity at 220 nm was observed, providing clear evidence for the in situ generation of H^+^ during the irradiation process.

**FIGURE 7:**
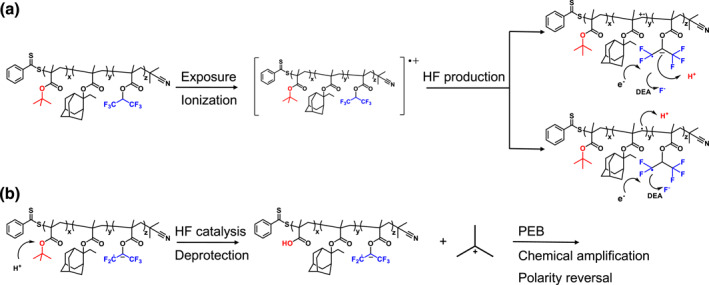
Proposed reaction mechanism of fluorinated polymer photoresists under electron beam irradiation. (a) Ionization‐induced dissociation, accompanied by C–F bond cleavage and subsequent formation of HF, (b) acid‐catalyzed deprotection with subsequent polarity reversal. HF, hydrofluoric acid.

On the basis of these results, the radiation‐induced lithographic mechanism of the PTAF copolymers is proposed (Figure 7). Upon irradiation, the –CF_3_ groups effectively capture low‐energy electrons, initiating the DEA process.[^4^, ^32^, ^33^, ^34^, ^35^, ^36^] This process leads to the cleavage of the C–F bond and results in the formation of transient anionic intermediates.[^37^, ^38^] These F^−^ ions abstract protons to form hydrofluoric acid, which, in conjunction with the nucleophilic attack of F^−^, significantly increases the local acid concentration. Subsequently, during the post‐exposure baking process, these photo‐generated acid catalysts effectively trigger the deprotection of the acid‐sensitive tBMA units. This deprotection causes the tBMA units to decompose into isobutylene and polar carboxyl groups. As a result, the polarity of the polymer in the exposed areas increases significantly, leading to a marked polarity reversal. Consequently, the exposed areas become insoluble in the non‐polar developing solution (*n*‐heptane), while the unexposed areas remain soluble. These multi‐step reaction processes enable the formation of high‐resolution negative‐tone lithographic patterns.

## 4 CONCLUSIONS

In summary, a series of novel fluorinated methacrylate‐based ternary copolymers (PTAF series) was successfully synthesized via controlled radical polymerization and systematically optimized for high‐performance photoresist applications. Under optimal conditions, including a 30 wt% solid content, 85°C polymerization temperature, 2.2 wt% chain transfer agent content, and a monomer molar ratio of 1:1:2, the copolymers exhibited narrow molecular weight distributions (PDI = 1.2–1.3), excellent thermal stability (*T*_g_ = 105°C, *T*_d_ initial = 212°C), and significantly enhanced hydrophobicity. When applied as a negative‐tone electron‐beam photoresist, PTAFIII achieved a high resolution of 16.5 nm at an exposure dose of 27.5 nC cm^−1^, together with low line edge roughness (LER = 2.9 nm) and good etch resistance. Mechanistic investigations confirm that C–F bond cleavage generates acid species that catalyze tert‐butyl deprotection and polarity reversal, which govern the solubility switching and pattern formation behavior. This work provides a feasible strategy for designing multifunctional methacrylate photoresists by integrating the synergistic advantages of adamantyl, fluorinated groups, and tert‐butyl ester groups. The as‐prepared PTAF copolymers exhibit significant application potential in advanced electron‐beam and EUV lithography, offering valuable guidance for the development of high‐performance polymer‐based photoresist materials.

## CONFLICT OF INTEREST STATEMENT

The authors declare no conflicts of interest.

## ETHICS STATEMENT

No animal or human experiments were involved in this study.

## Supporting information

Supporting Information S1

## Data Availability

The data that supports the findings of this study are available in Supporting Information S1 of this article.
