# Supplementary material for: Fluorinated copolymer resists for high‐resolution negative‐tone lithography: Preparation and patterning performance
Source: Smart Mol. 2026 May 28:e70062. Online ahead of print. doi: 10.1002/smo2.70062 (PMC13398754; doi:10.1002/smo2.70062)
Supplement: Supplementary file 1 — Supporting Information S1 [file SMO2-9999-0-s001.docx]

**Supporting Information**

**Fluorinated Copolymer Resists for High-Resolution Negative-tone Lithography: Preparation and Patterning Performance**

Yuting Tang^1^, Wenzheng Li^1^, Xinyan Huang^1^, Min Zhang^1^, Han Han^1^, Jialong Zhang^2,^*, Qinyu Luo^2^, Jiangli Fan^1^, Pengzhong Chen^1,^*, and Xiaojun Peng^1^

^1^State Key Laboratory of Fine Chemicals, Frontiers Science Center for Smart Materials, Dalian University of Technology, Dalian 116024, China;

^2^Sinopec Shanghai Research Institute of Petrochemical Technology Co., Ltd., Shanghai 201208, China

CONTENT

[1 Synthesis of compounds 1](#_Toc227690045)

[2 Molecular structure characterization 3](#_Toc227690046)

[3 GPC test results 5](#_Toc227690047)

[4 FT-IR absorption spectra 6](#_Toc227690048)

[5 UV–vis absorption spectra 6](#_Toc227690049)

[6 AFM test results 7](#_Toc227690050)

[7 EBL test results 8](#_Toc227690051)

[8 XPS test results 10](#_Toc227690052)

[9 UV–vis absorption spectra before and after exposure 11](#_Toc227690053)

# Synthesis of compounds


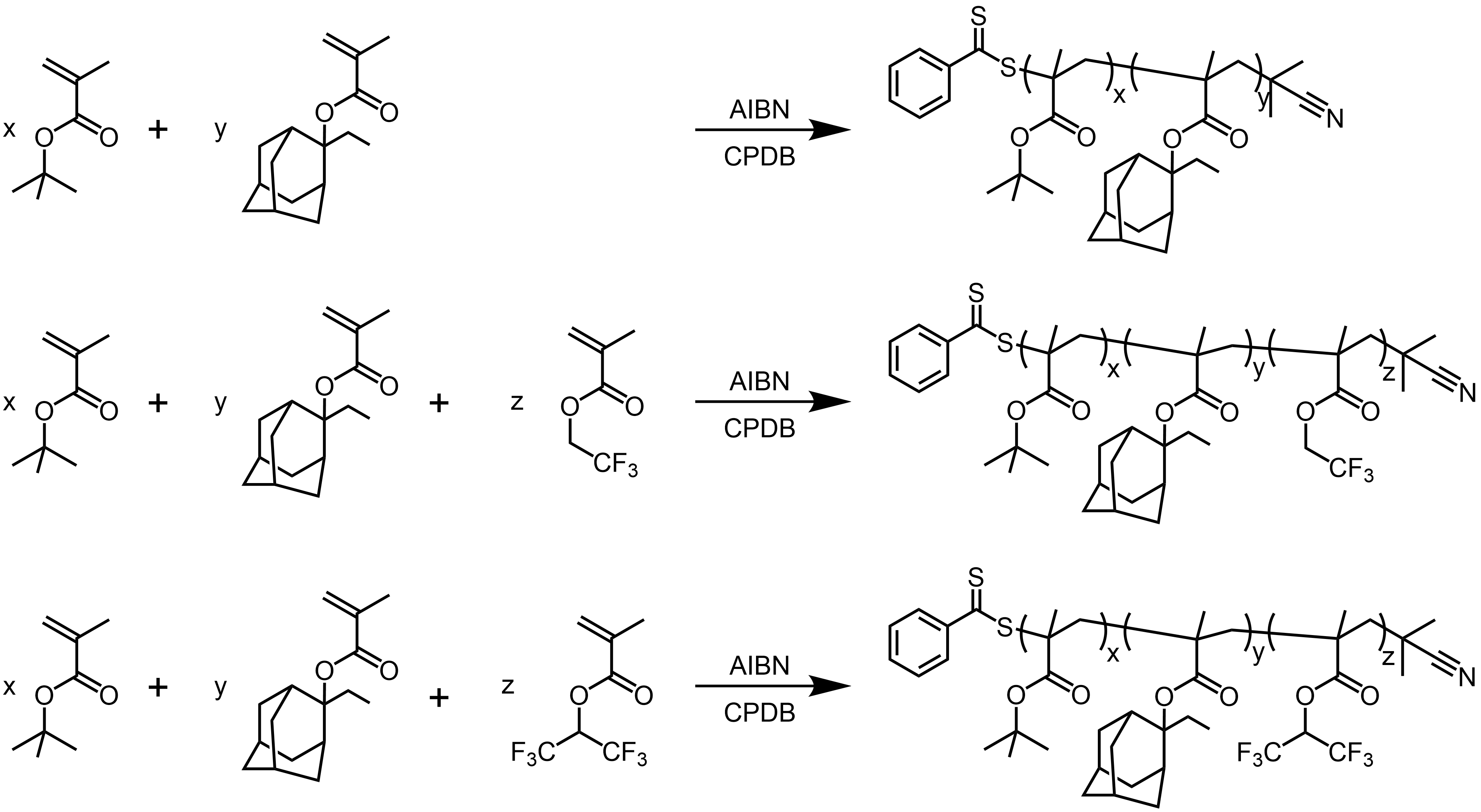


Scheme S1: The synthetic pathway for the compounds.

**Synthesis of PTAⅠ**

Poly (tert-butyl methacrylate-co-2-ethyl-2-methylacryloyloxy-pentane) (p(tBMA-co-EAdMA), denoted as PTAⅠ) is described as follows. tBMA (0.569 g, 4 mmol), EAdMA (0.993 g, 4 mmol), were dissolved in 4 mL of DMF together with the initiator AIBN (0.017 g, 1.1 wt% relative to the total monomer mass) and the chain transfer agent CPDB (0.034 g, 2.2 wt% relative to the total monomer mass). After degassing with nitrogen, the reaction mixture was heated to 85 °C and stirred for 12 h under a nitrogen atmosphere. Subsequently, to quench the residual chain transfer agent, a 70 wt% AIBN solution (0.81 g AIBN in 2 mL DMF) was added, and the system was held at 60 °C for another 12 h. After reaction, the polymer solution was slowly dropped into 500 mL of deionized water to precipitate the product. The resulting precipitate was collected by filtration and vacuum-dried at 45 °C for 24 h to obtain purified PTAⅠ. The yield is 75%.

^1^H NMR (500 MHz, CDCl_3_): δ 2.41 (s, 1H), 2.26–1.91 (m, 2H), 1.71 (s, 10H), 1.63–0.73 (m, 8H).

**Synthesis of PTAFII**

Poly (tert-butyl methacrylate-co-2-ethyl-2-methylacryloyloxy-pentane-co-trifluoroethyl methacrylate) (p(tBMA-co-EAdMA-co-TFEMA), denoted as PTAFII) is described as follows. tBMA (0.569 g, 4 mmol), EAdMA (0.993 g, 4 mmol), and TFEMA (1.34 g, 8 mmol) were dissolved in 7.4 mL of DMF together with the initiator AIBN (0.032 g, 1.1 wt% relative to the total monomer mass) and the chain transfer agent CPDB (0.064 g, 2.2 wt% relative to the total monomer mass). After degassing with nitrogen, the reaction mixture was heated to 85 °C and stirred for 12 h under a nitrogen atmosphere. Subsequently, to quench the residual chain transfer agent, a 70 wt% AIBN solution (1.5 g AIBN in 4.2 mL DMF) was added, and the system was held at 60 °C for another 12 h. After reaction, the polymer solution was slowly dropped into 500 mL of deionized water to precipitate the product. The resulting precipitate was collected by filtration and vacuum-dried at 45 °C for 24 h to obtain purified PTAFII. The yield is 72%.

^1^H NMR (400 MHz, CDCl_3_): δ 4.35 (s, 1H), 2.32 (s, 4H), 1.72 (s. 9H), 1.42 (d, *J* = 7.1 Hz, 4H), 1.01 (t, *J* = 37.7 Hz, 3H).

**Synthesis of PTAFⅢ**

Poly (tert-butyl methacrylate-co-2-ethyl-2-methylacryloyloxyadamantane-co-1,1,1,3,3,3-hexafluoroisopropyl isobutyrate methacrylate) (p(tBMA-co-EAdMA-co-HFIPMA), denoted as PTAFⅢ) is described as follows. tBMA (0.569 g, 4 mmol), EAdMA (0.993 g, 4 mmol), and HFIPMA (1.89 g, 8 mmol) were dissolved in 8 mL of DMF together with the initiator AIBN (0.038 g, 1.1 wt% relative to the total monomer mass) and the chain transfer agent CPDB (0.076 g, 2.2 wt% relative to the total monomer mass). After degassing with nitrogen, the reaction mixture was heated to 85 °C and stirred for 12 h under a nitrogen atmosphere. Subsequently, to quench the residual chain transfer agent, a 70 wt% AIBN solution (1.7 g AIBN in 4.8 mL DMF) was added, and the system was held at 60 °C for another 12 h. After reaction, the polymer solution was slowly dropped into 500 mL of deionized water to precipitate the product. The resulting precipitate was collected by filtration and vacuum-dried at 45 °C for 24 h to obtain purified PTAFⅢ. The yield is 82%.

^1^H NMR (400 MHz, CDCl_3_): δ 5.68 (s, 1H), 2.31 (s. 2H), 2.05 (d, *J* = 49.5 Hz, 8H), 1.72 (d, *J* = 1.2 Hz, 19H), 1.43 (s, 8H), 1.31–0.72 (l, 7H).

# Molecular structure characterization


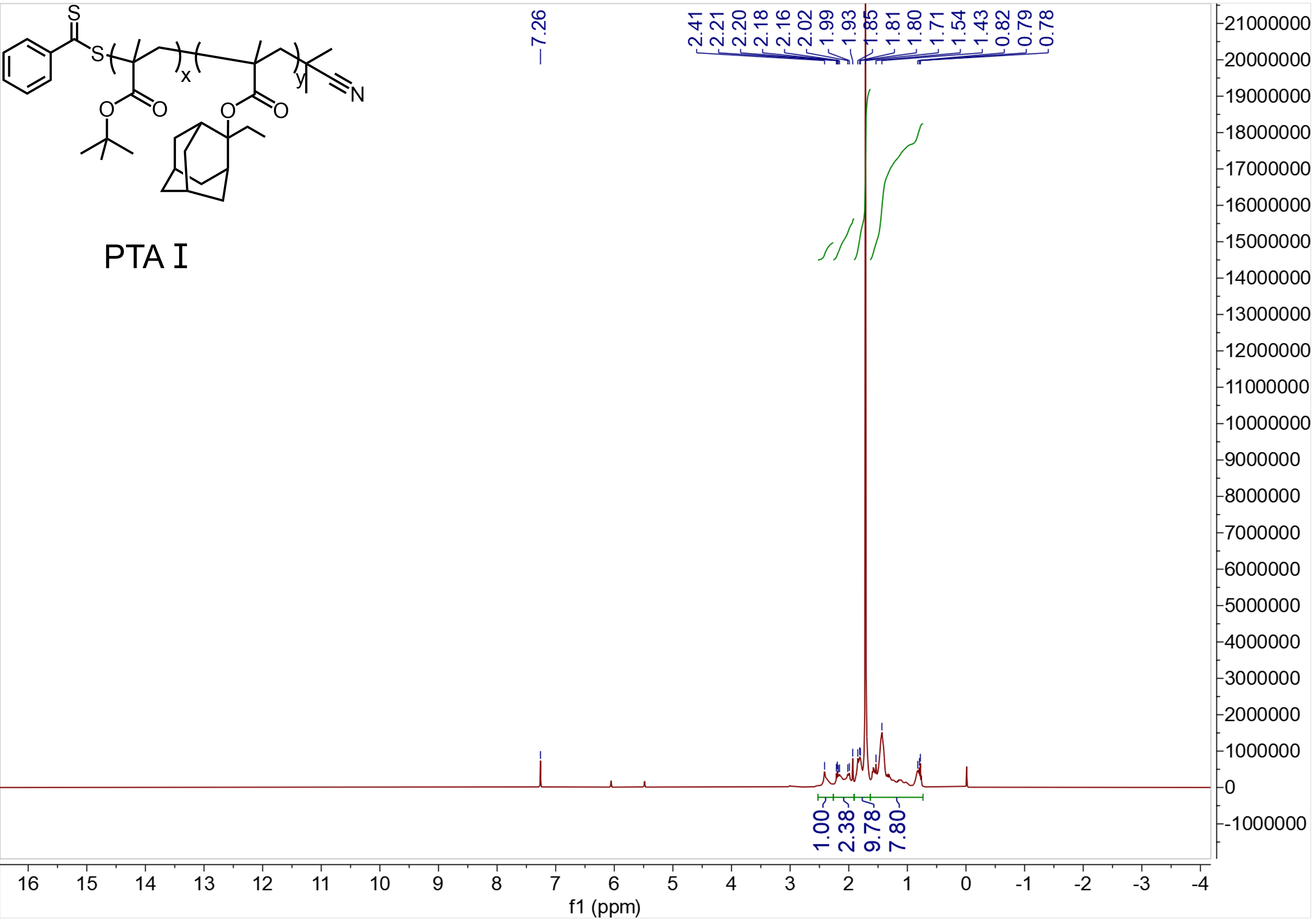


**Figure S1.** ^1^H NMR spectrum of **PTAⅠ** in CDCl_3_.

**
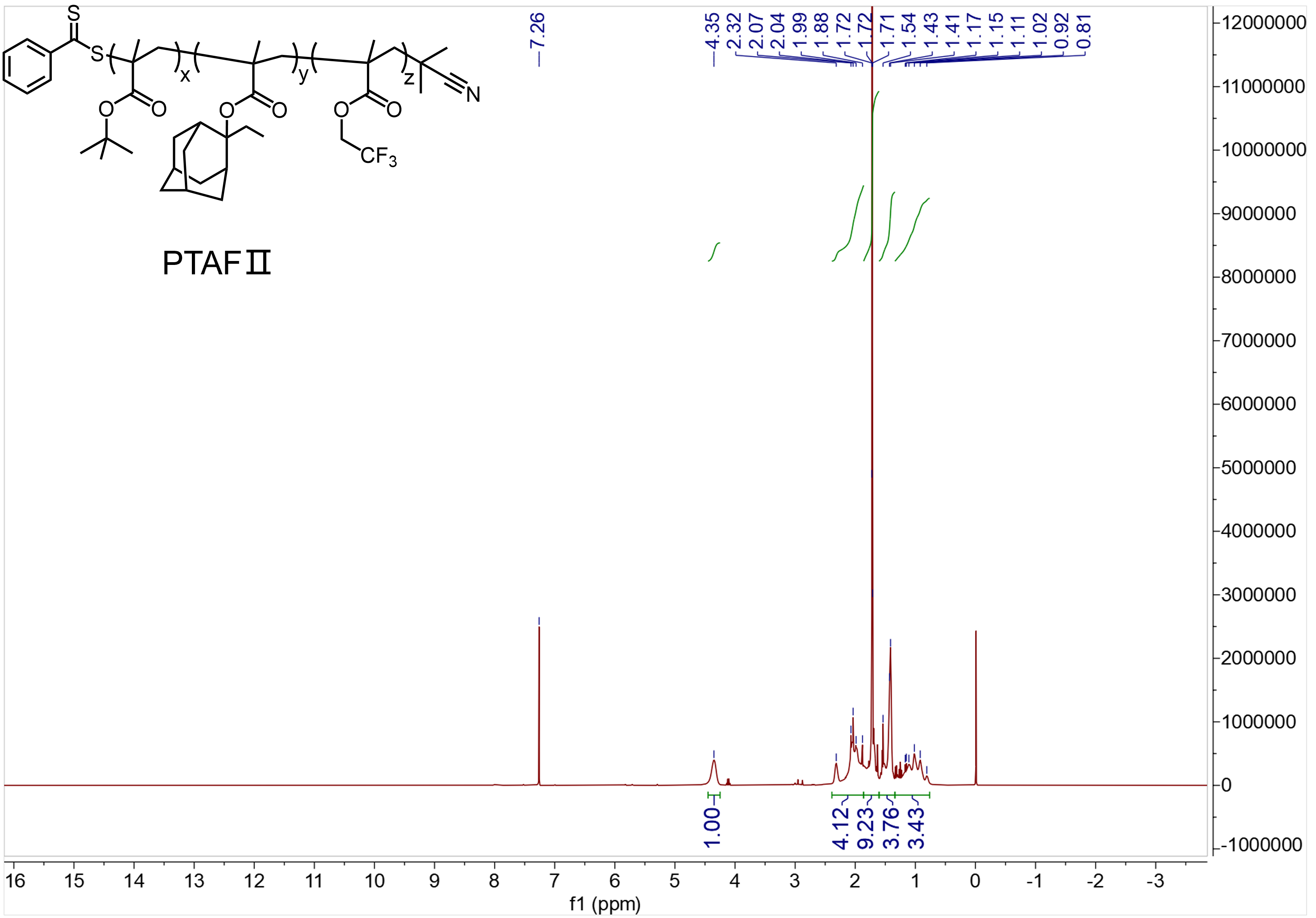
**

**Figure S2.** ^1^H NMR spectrum of **PTAFⅡ** in CDCl_3_.


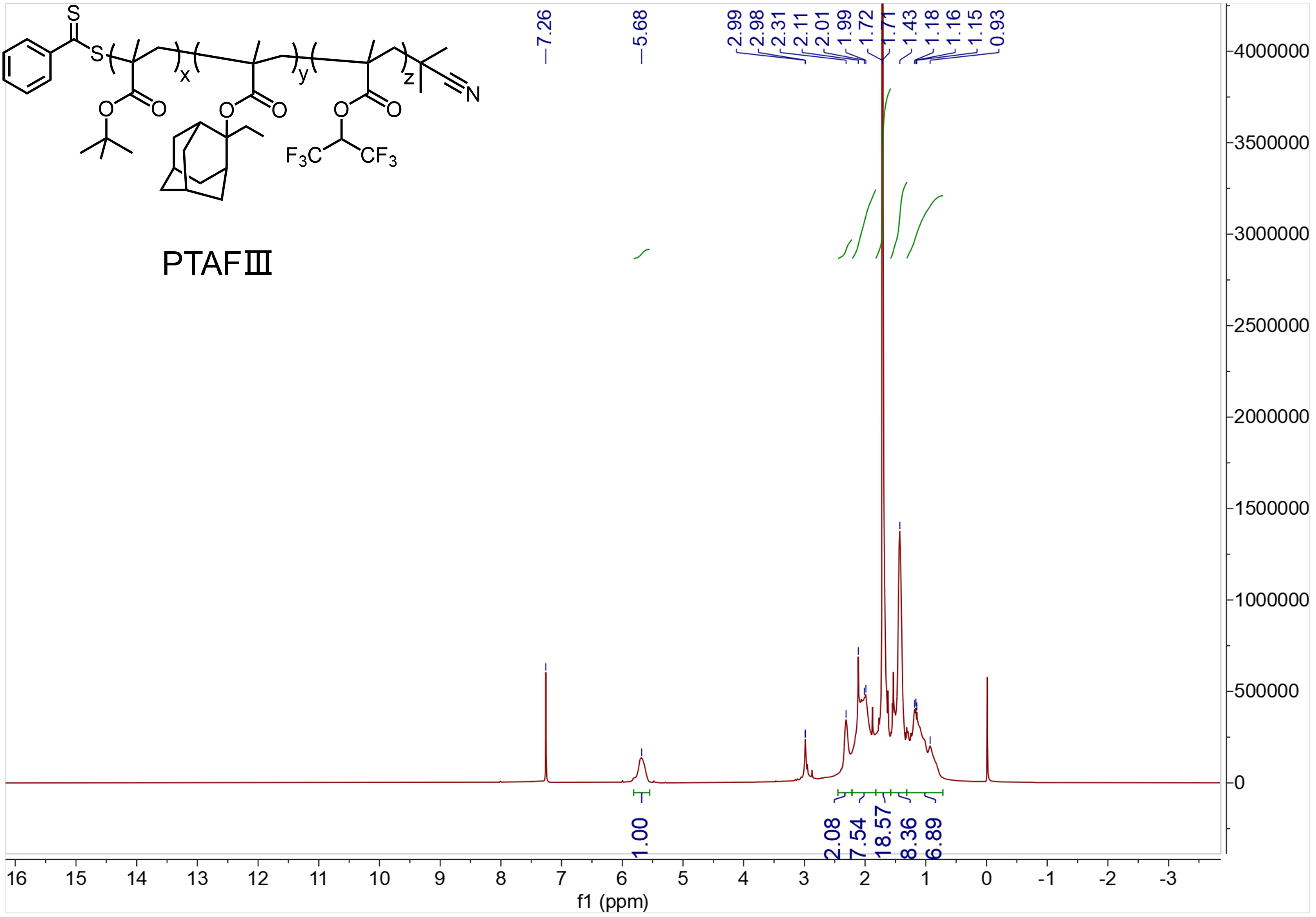


**Figure S3.** ^1^H NMR spectrum of **PTAFⅢ** in CDCl_3_.

# GPC test results

**Table S1**. M_n_, M_w_ and PDI at different solid contents.

| Solid content (wt%) | M_n_ (g mol^-1^) | M_w_ (g mol^-1^) | PDI |
| --- | --- | --- | --- |
| 50 | 9917 | 14632 | 1.475 |
| 40 | 9344 | 13744 | 1.471 |
| 30 | 9052 | 13068 | 1.444 |
| 20 | 8210 | 11875 | 1.446 |
| 10 | 6318 | 9229 | 1.461 |

**Table S2.** M_n_, M_w_ and PDI at Different Temperatures.

| Temperature (℃) | M_n_ (g mol^-1^) | M_w_ (g mol^-1^) | PDI |
| --- | --- | --- | --- |
| 70 ℃ | 14483 | 26093 | 1.802 |
| 85 ℃ | 9052 | 13068 | 1.444 |
| 100 ℃ | 7624 | 11744 | 1.540 |

**Table S3.** M_n_, M_w_ and PDI at different CPDB contents.

| CPDB (wt%) | M_n_ (g mol^-1^) | M_w_ (g mol^-1^) | PDI |
| --- | --- | --- | --- |
| 1.1 | 7624 | 26093 | 1.802 |
| 2.2 | 5355 | 7181 | 1.341 |
| 3.3 | 5534 | 7947 | 1.436 |

**Table S4.** M_n_, M_w_ and PDI at different monomer ratios.

| tBMA: EAdMA: HFIPMA | M_n_ (g mol^-1^) | M_w_ (g mol^-1^) | PDI |
| --- | --- | --- | --- |
| 1:1:1 | 4163 | 5588 | 1.342 |
| 1:2:1 | 3832 | 4970 | 1.297 |
| 1:1:2 | 4271 | 5435 | 1.272 |

# FT-IR absorption spectra


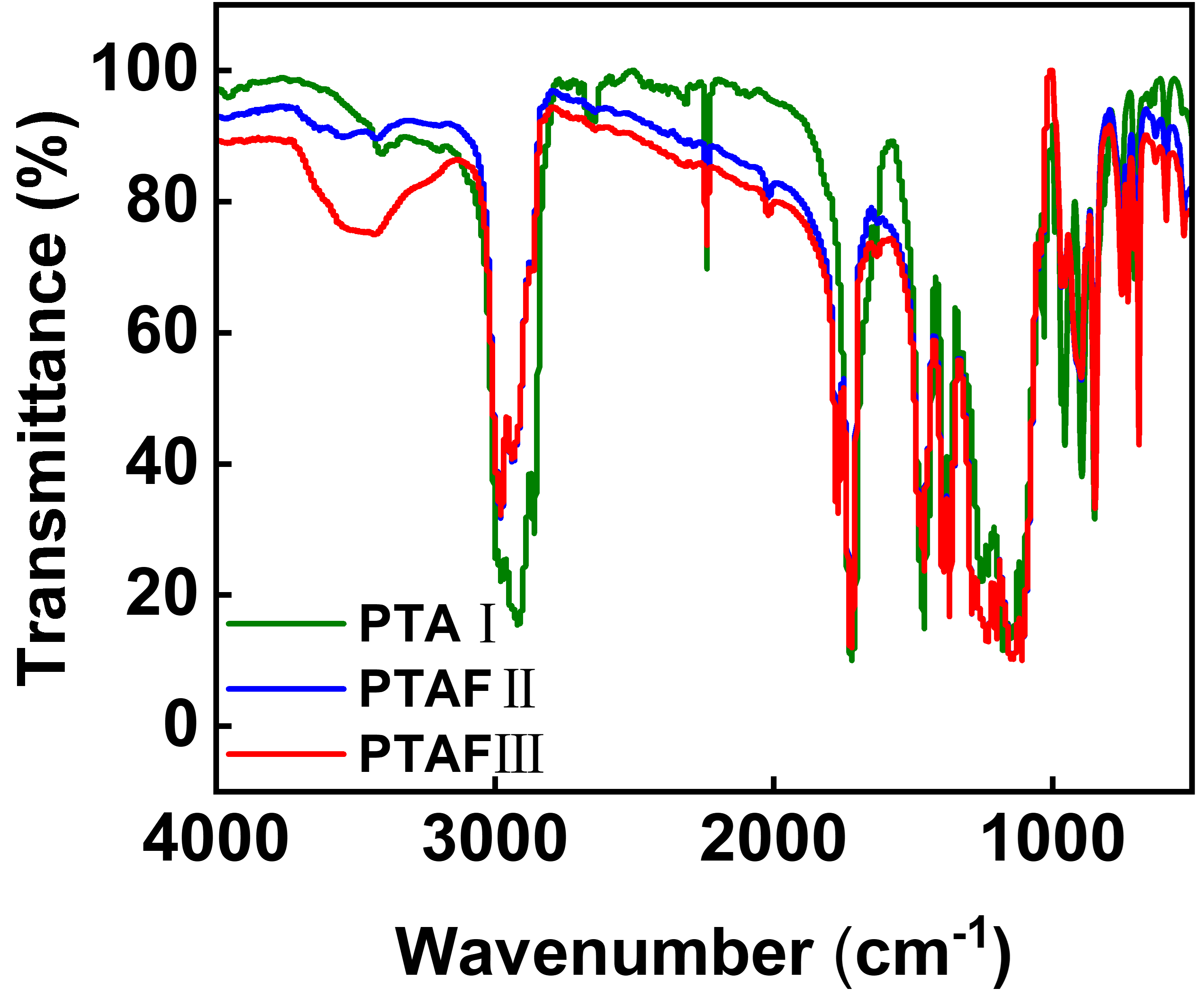


**Figure S4**. FT-IR spectra of **PTAI, PTAFII,** and **PTAFIII**.

# UV–vis absorption spectra


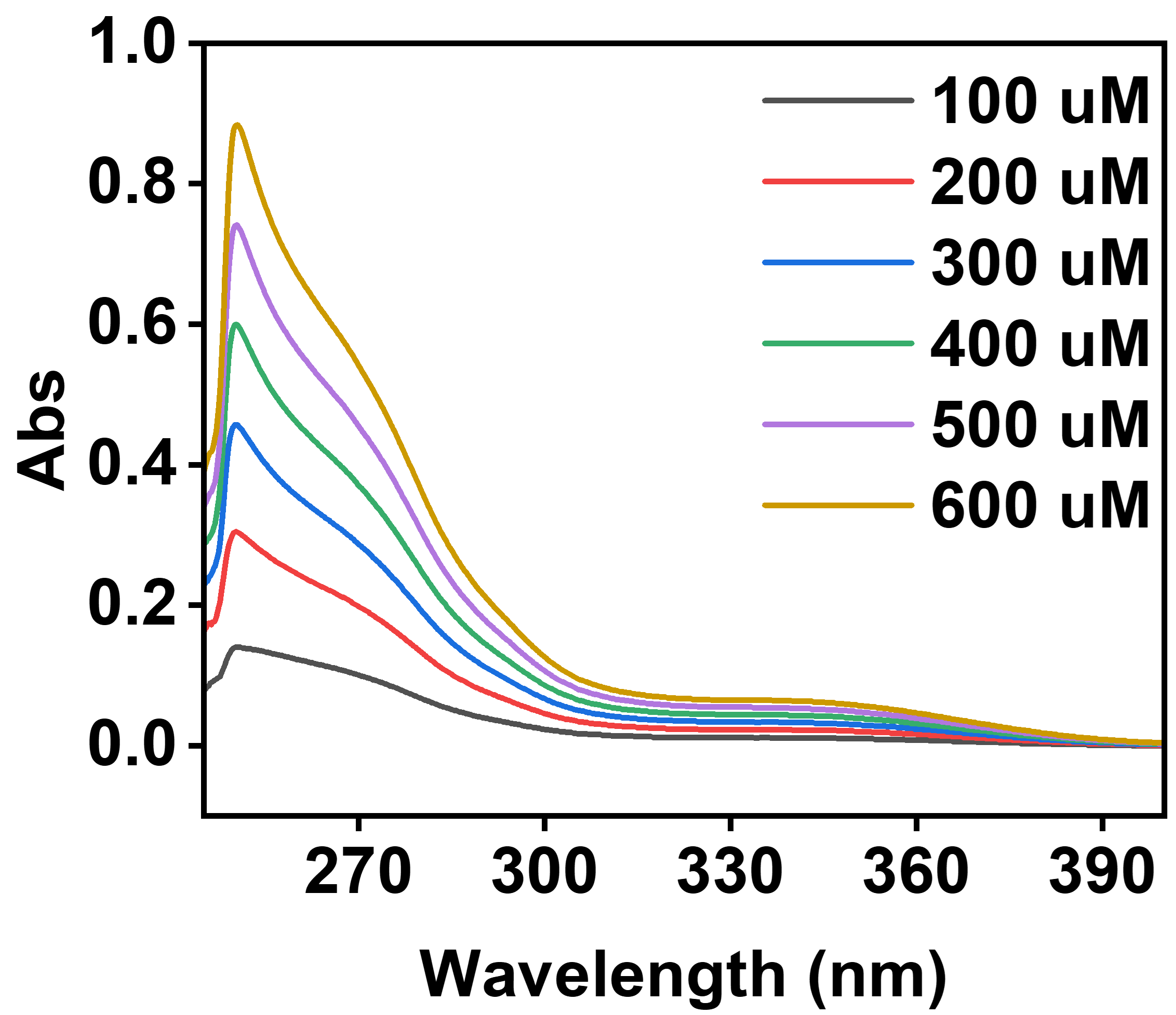


**Figure S5**. The UV–vis absorption spectra of **PTAFⅢ** in acetonitrile at 298 K.

# AFM test results


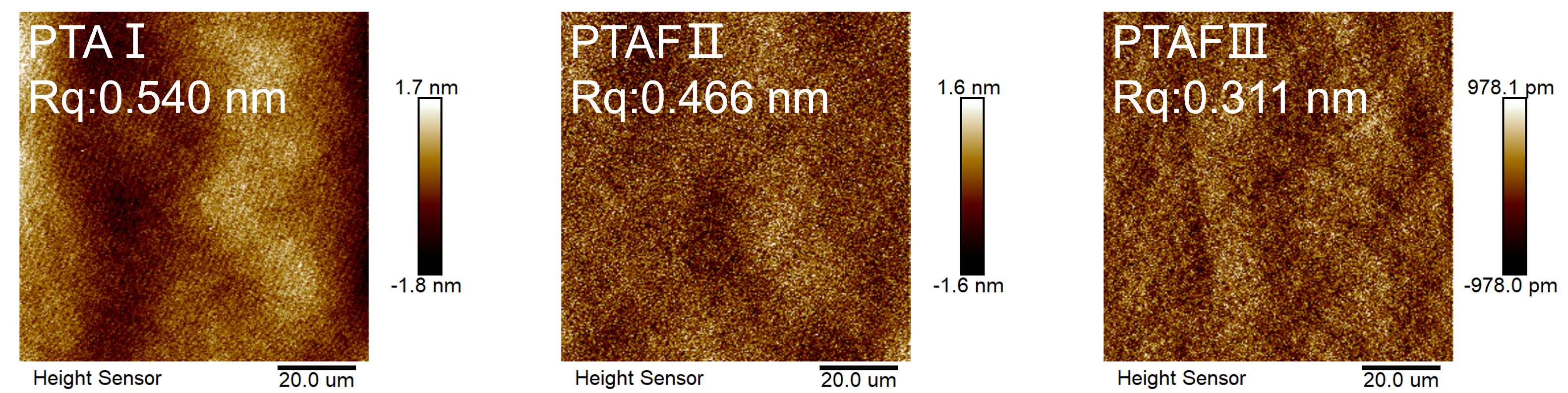


**Figure S6**. The AFM 2D morphology images of **PTAⅠ, PTAFⅡ, PTAFⅢ**.


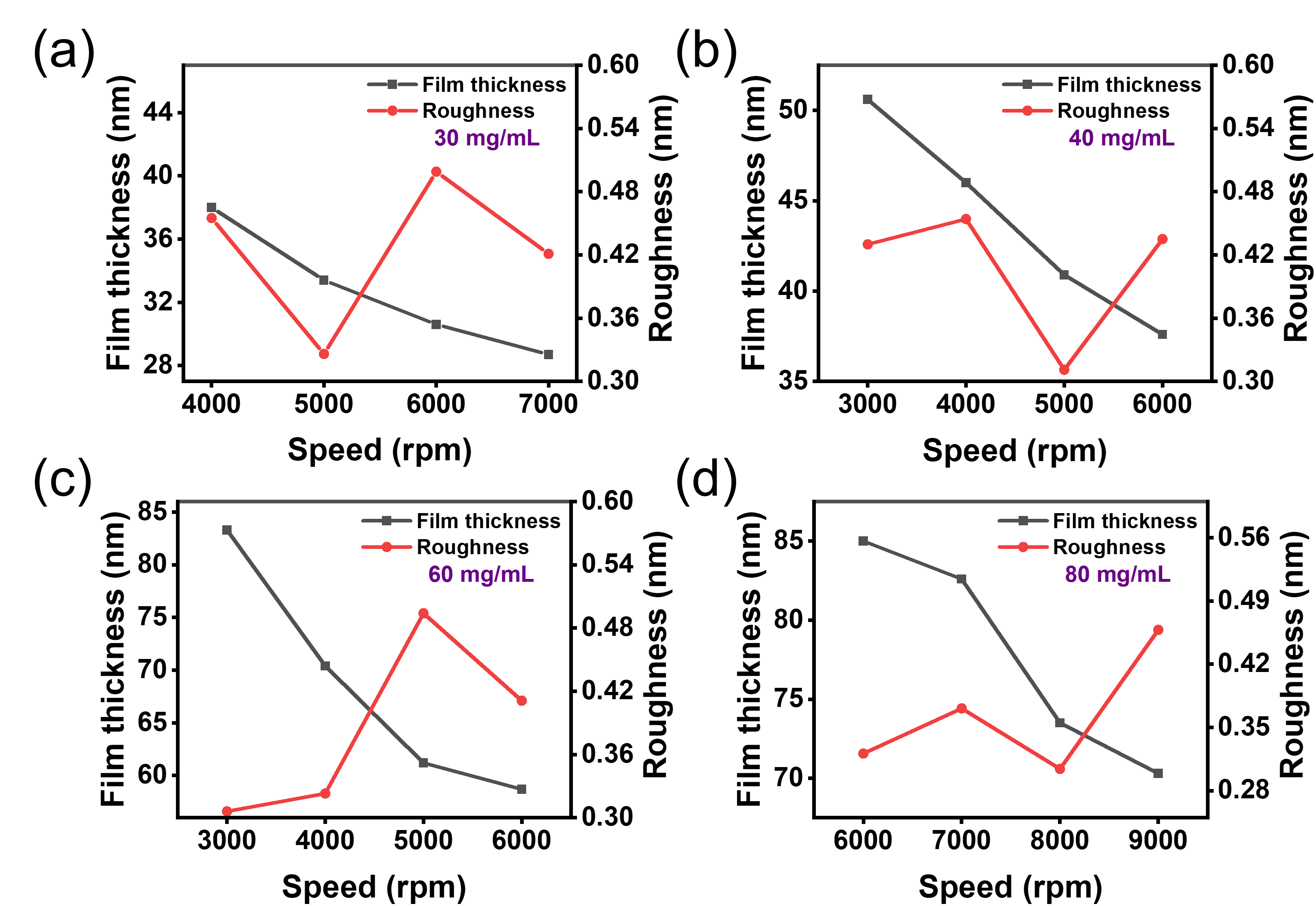


**Figure S7**. The effect of rotational speed on surface roughness and film thickness of **PTAFⅢ** at different concentrations.

# EBL test results


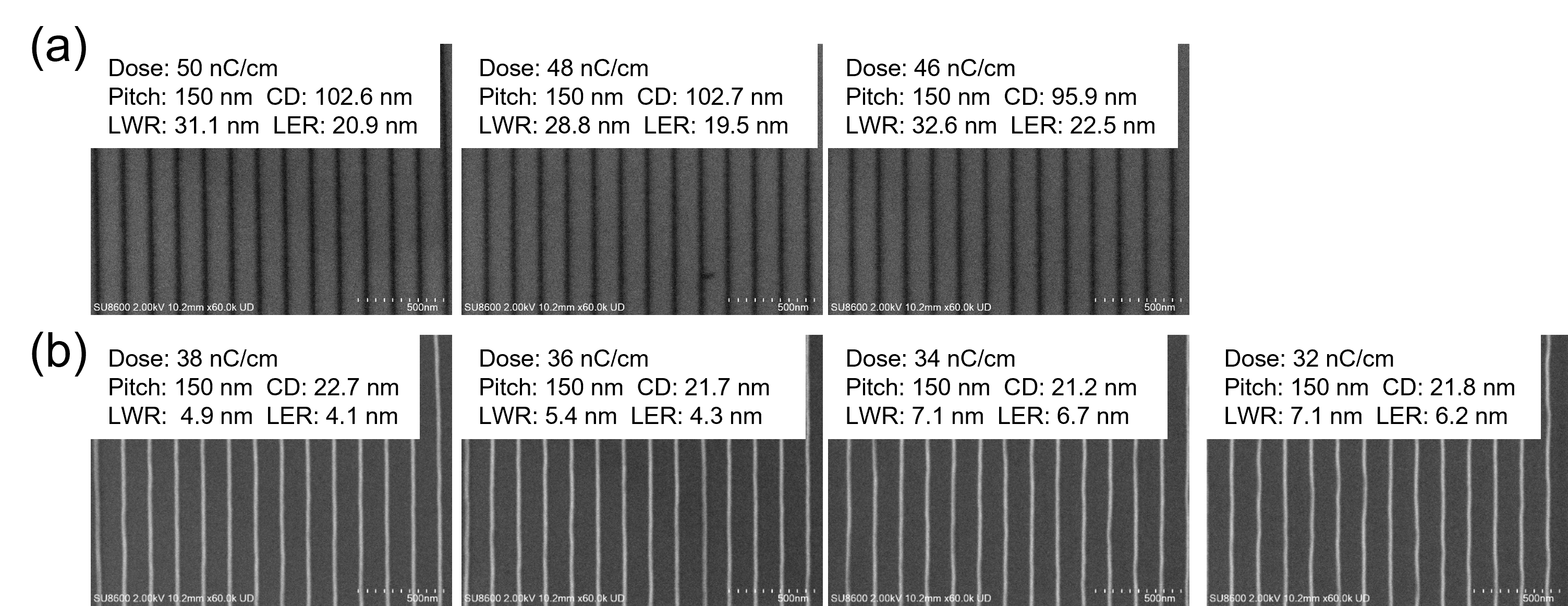


**Figure S8**. SEM images at different concentrations. (a) 20 mg mL^-1^, (b) 40 mg mL^-1^.


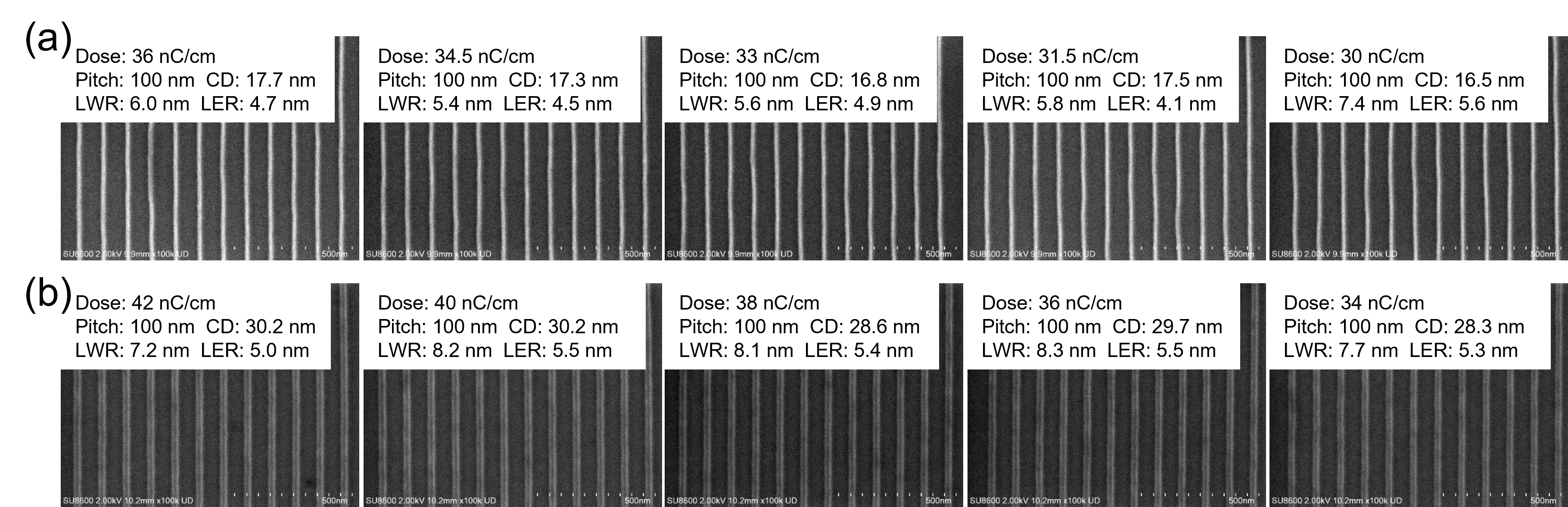


**Figure S9**. SEM images at different pre-application bake temperatures. (a) 50 ℃, (b) 70 ℃.


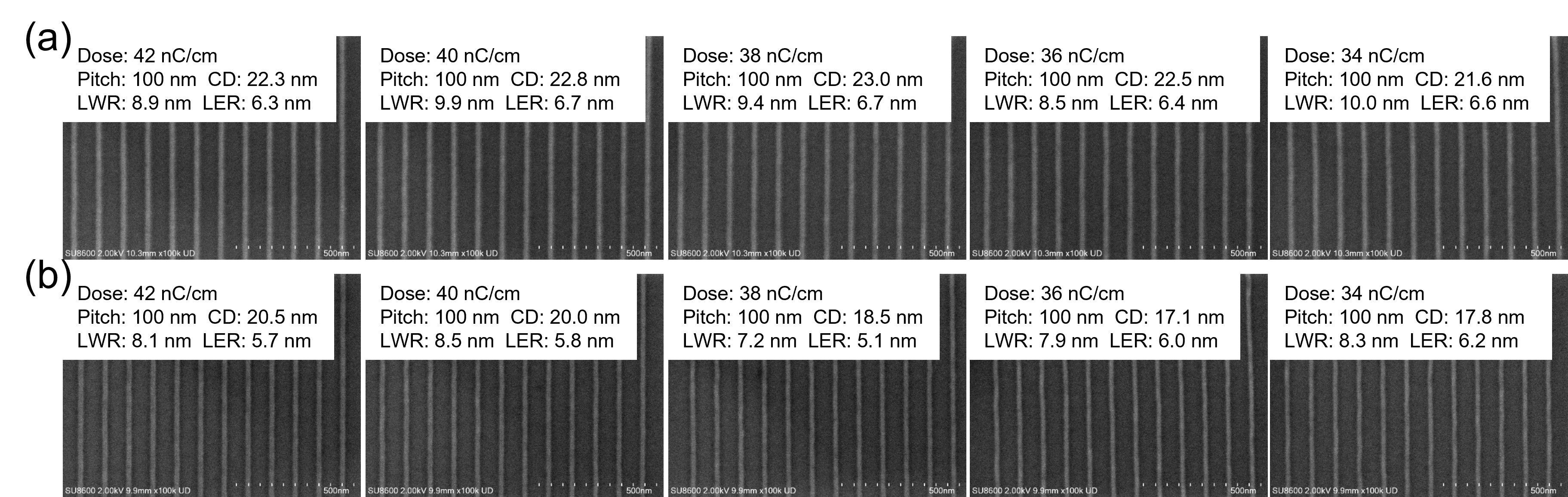


**Figure S10**. SEM images at different spin-coating speeds. (a) 4000 rpm, (b) 6000 rpm.


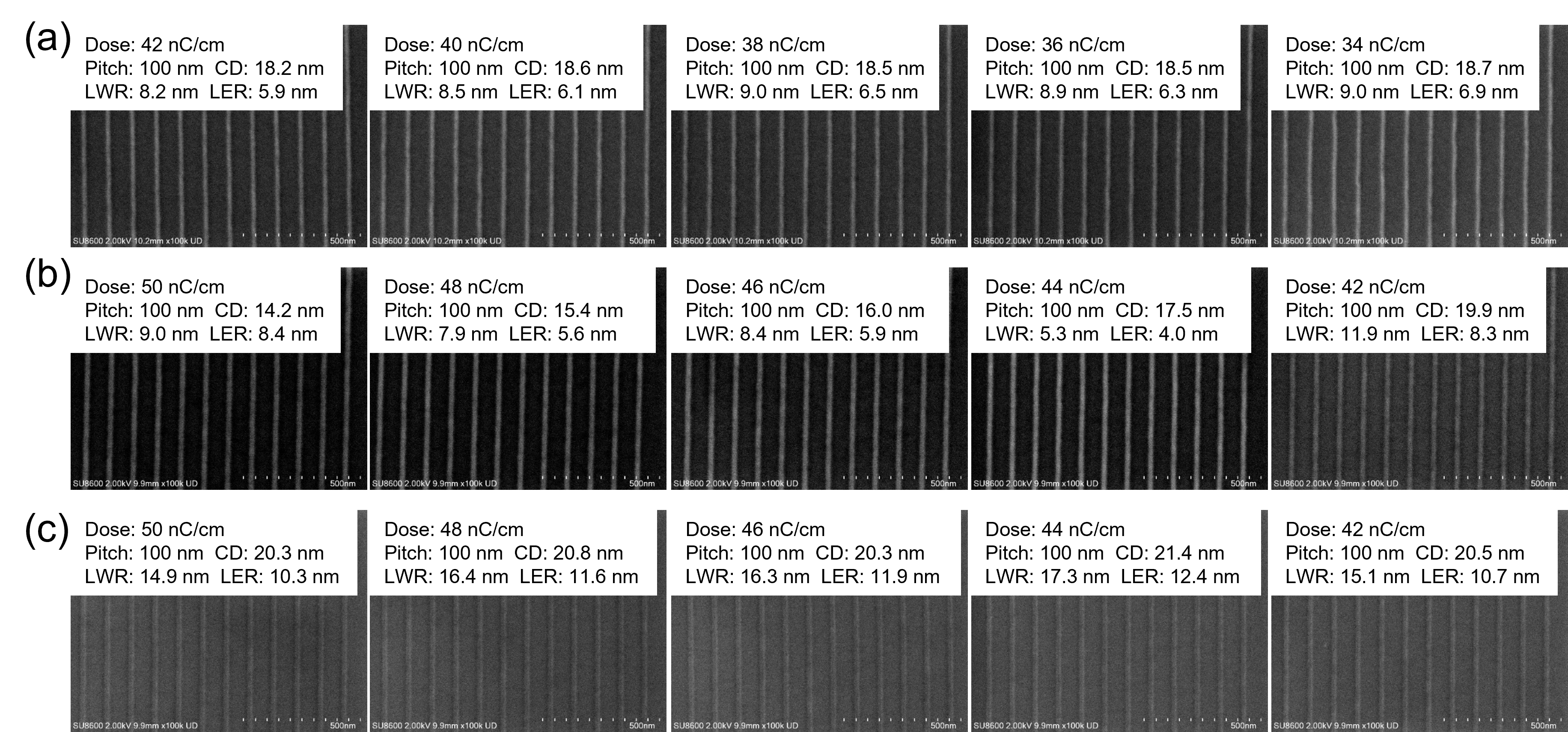


**Figure S11**. SEM images at different post-exposure bake temperatures. (a) 50 ℃, (b) 70 ℃, (c) 90 ℃.

# XPS test results

**Figure S12**. XPS characteristic spectra of fluoromethacrylate-based photoresists under 254 nm UV light with different exposure times: (a) C 1s spectra, (b) F 1s spectra, (c) O 1s spectra (exposure times are 0, 5, 10, 30 min, respectively).

**Table S5.** Atomicpercentages of C, O, and F in the unexposed and exposed resist films.

| Element | Atomic% | | | | △Atomic% |
| --- | --- | --- | --- | --- | --- |
|  | Unexposed | Exposed 5 min | Exposed 10 min | Exposed 30 min |  |
| C(total) | 69.68 | 68.93 | 67.31 | 59.08 | -10.6 |
| C–C/C–H | 31.52 | 40.58 | 33.47 | 32.50 | 0.98 |
| C–O | 24.10 | 7.59 | 19.15 | 10.29 | -13.81 |
| C=0 | 10.14 | 16.93 | 12.26 | 14.67 | 4.53 |
| F(total) | 15.15 | 15.08 | 13.58 | 7.33 | -7.82 |
| C–F | 3.92 | 3.83 | 2.43 | 1.62 | -2.30 |
| O(total) | 15.17 | 15.99 | 19.11 | 33.59 | 18.42 |
| O–C | 9.83 | 10.01 | 15.19 | 3.02 | -6.81 |
| O=C | 5.34 | 5.98 | 3.92 | 30.57 | 25.23 |

# UV–vis absorption spectra before and after exposure

**Figure S13.** UV–vis absorption spectra of a film composed of PTAFⅢ and the pH indicator bromophenol green before and after exposure to UV light.
